# Drugs against osteoporosis in perimenopausal and postmenopausal women

**DOI:** 10.3389/fmed.2025.1682333

**Published:** 2025-11-13

**Authors:** Hong Lv, Yaoyang Zhang, Jiaqian Li, Qiucheng Jia, Yi Yu, Huimin Tang, Wanying Chen, Yun Yang, Qiong Jiang, Jiming Chen

**Affiliations:** 1 People’s Hospital of Pujiang County, Chengdu, China; 2 Department of Gynecology, The Second People’s Hospital of Changzhou, The Third Affiliated Hospital of Nanjing Medical University, Changzhou, China; 3 Department of Obstetrics and Gynaecology, Longgang District People’s Hospital, Shenzhen, China; 4 Jiangxi Medical College, Shangrao, China; 5 Department of Gynecology, Shiyan Hospital of Traditional Chinese Medicine, Shiyan, China

**Keywords:** perimenopause, menopause, osteoporosis, drug therapy, BMD

## Abstract

The prevalence of osteoporosis will continue to rise as the world’s population ages. Since women’s bones are smaller and thinner than men’s, bone loss accelerates as estrogen levels fall. Based on their primary effects, drugs used to treat osteoporosis can be categorized into three groups: 1. Anti-resorptive medications. These primarily prevent osteoclasts from resorbing bone, which has a secondary effect on bone growth. Commonly used anti-resorptive drugs include bisphosphonates, denosumab, hormone replacement therapy, and raloxifene. 2. Anabolic medicines. These mainly stimulate osteoblasts to produce new bone, although they may also affect bone resorption. Common examples include teriparatide, romosozumab, and parathyroid hormone. 3. Phytoestrogens. These are non-steroidal, naturally occurring plant compounds with estrogenic and/or anti-estrogenic properties that resemble estrogen.

## 1 Introduction

Increased bone fragility and a heightened risk of fracture are the outcomes of osteoporosis, a progressive systemic skeletal disease marked by a loss in bone mass and microstructural degradation of bone tissue (1). Approximately 200 million people worldwide suffer from osteoporosis. In China, there were 411,000 hip fracture cases in 2015, projected to rise to 1 million by 2050 (2). This disease imposes a heavy burden on healthcare systems. Data indicates that medical expenses for osteoporotic fractures are projected to reach 132 billion yuan by 2035, with the burden increasing as the proportion of elderly individuals grows (2). During perimenopause (MT), women’s estrogen levels drop. This causes an increase in osteoclast resorption activity without a corresponding increase in osteoblast activity. This disrupts the normal cycle of bone metabolism, causing more bone to be reabsorbed than deposited and a net loss of bone. The SWAN (Study of Women’s Health Across the Nation) prospective cohort study documented a pattern of declining bone mineral density (BMD) during MT (perimenopause), with an accelerating trend beginning 1 year before menopause and continuing for 3 years, followed by a moderate rate of bone loss over the next four to 8 years (3). The average decline in BMD during the MT is about 10%, with bone loss of up to 20% during the 5–7 years around the menopause (4). The current recommendations for the pharmacologic treatment of osteoporosis state that a woman may be eligible for treatment if she has a history of fragility fractures, a dual-energy X-ray absorptiometry (DEXA) indicates a BMD T-score of < −2.5, or a T-score between −1 and −2.5 indicates a high risk of fracture (5).

## 2 Primary therapeutic approach

### 2.1 Bisphosphonates

During bone resorption, osteoclasts absorb bisphosphonates. In osteoclasts, aminobisphosphonates (e.g., alendronate, risedronate, zoledronate) impair the resorption capacity and cellular integrity of osteoclasts and also trigger apoptosis. Also bisphosphonates cause a progressive increase in BMD, which is more pronounced in trabeculae-rich sites (e.g., spine) (6). Women older than 60 years old may benefit most from bisphosphonates in preventing and treating osteoporosis. It is recommended that alendronate 70 mg be administered once weekly to prevent and treat postmenopausal osteoporosis (PMO). Women with low BMD of the femoral neck, aged 55–81, were randomly assigned to one of two study groups based on whether or not they were currently experiencing a vertebral fracture. A total of 2,027 women were followed for 36 months after being randomly assigned to either the placebo (1,005) or the alendronate (1,022) groups). Alendronate was effective in reducing the incidence of vertebral fracture compared with placebo (7). In another randomized double-blind controlled trial, women aged 54–81 years with femoral neck BMD ≤ 0.68 g/cm^2^ and no vertebral fractures. Women without spinal fractures and between the ages of 54 and 81 with a femoral neck BMD of ≤0.68 g/cm^2^. Alendronate or a placebo was given to them randomly; the alendronate group had a dose of 5 mg/d for 2 years, then an adjusted dose of 10 mg/d for the remaining 2 years. The alendronate group showed an increase in BMD at all study locations, and among patients with baseline femoral neck osteoporosis, there was a 36% reduction in clinical fractures (8). Note that only patients with normal serum vitamin D levels and an estimated creatinine clearance of more than 35 milliliters per minute should be administered bisphosphonates. The main adverse reaction is digestive tract symptoms. Approximately 20–30% of patients taking oral bisphosphonates may experience upper gastrointestinal issues, such as gastroesophageal reflux (8). To minimize this risk, medical experts recommend that patients remain upright for at least 30 min after administration. Alternatively, intravenous formulations of bisphosphonates can avoid these problems (Table 1).

**Table 1:** The main drugs for the prevention and treatment of osteoporosis in perimenopausal and postmenopausal women.

		Drugs	Dosage	Indications	Adverse reactions	Precautions
Antiresorptive	Bisphosphonates	Alendronate	70 mg orally once weekly	Female, aged 60 years and older, femoral neck	Gastrointestinal symptoms, such as gastroesophageal reflux, may be appropriately relieved by remaining upright for at least 30 min after dosing	Patients with creatinine clearancegreater than 35 mL per minute and normalserum vitamin Dlevels
		Zoledronate	5 mg, IV infusion, once a year	PMO and postmenopausal women, vertebral fractures, hip fractures, nonvertebral fractures	Fever, musculoskeletal pain and discomfort. Acetaminophen may be used 2. Vitamin D deficiency, prone to hypocalcemia	Evaluate serum 25-hydroxyvitamin D, calcium, phosphorus, and urinary calcium excretion levels to obtain adequate calcium and vitamin D intake
		Denosumab	60 mg subcutaneously every 6 months	Vertebral, nonvertebral, and hip fractures in postmenopausal women with osteoporosis	Osteosarcoma has occurred inlong-term and high-dose administration, and two rare adverse reactions have been associated with long-term administration of bisphosphonate and denosumab: osteonecrosis of the jaw and atypical femur fracture.	Bone loss worsens after stopping the drug and can be prevented by switching to another anti-bone resorption drug
	Estrogen replacement therapy (MHT)	Estradiol	0.014 mg/day orally	For women 50 years of age and younger, vertebral, hip, nonvertebral	1. monoestrogen increases the risk of endometrial hyperplasia and cancer, requiring the addition of progestin. 2. nausea, bloating, weight gain, fluid retention, mood swings, breakthrough bleeding (for the first 3–6 months), headache, and breast tenderness.	Use with caution in patients with unexplained vaginal bleeding, severe active liver disease, prior estrogen-sensitive breast or endometrial cancer, coronary artery disease, stroke, personal history of dementia or hereditary high risk of thromboembolism, and hypertriglyceridemia.
	Selective estrogen receptor modulator (SERM)	Raloxifene	60 mg/d	Women in the age group 50–59 years, vertebrae	Venous thromboembolism risk tripled, but breast cancer risk reduced	None
Bone formation stimulators	Agonistsof parathyroid hormone type 1 receptors	Teriparatide	20 μg/day subcutaneously for 18–24 months	Postmenopausal, glucocorticoid osteoporosis treatment, and patients at high risk of fracture	Nausea and headache were common at doses greater than 20 μg/day. Mild, transient, post-administration hypercalcemia was seen in a small percentage of patients.	There may be a risk of osteosarcoma development with very high doses of teriparatide, and the duration of treatment is limited to 24 months with subsequent antiresorptive medications.
	anti-sclerostin antibody	Romolizumab	210 mg/month subcutaneously for 12 months	Severe osteoporosis in postmenopausal women at very high risk of fracture, new vertebral, nonvertebral fractures	cardiac ischemic and cerebrovascular events, and should not be initiated in patients who have had a myocardial infarction or stroke within the previous year	None
Phytoestrogens	Isoflavones	Genistein Flavonoids, Epiflavonoids	Genistein 54 mg/d, Epiflavone 600 mg/d	Lumbar spine, femoral neck, and distal radius	Hair loss risk and central nervous system side effects: headache, drowsiness, dizziness, depression	Treatment course requires ≥24 months; efficacy of isoflavone extracts and dietary isoflavones is uncertain
	Black sesame	Black Cohosh Extract: BNO1055	40 mg/d	Unknown	Rare adverse reactions: gastrointestinal symptoms, allergic skin reactions, facial or peripheral edema, elevated serum liver enzymes, weight gain	Ingredients have many influencingfactors and unstable effects.

Postmenopausal women with osteoporosis are given 150 mg of ibandronate orally once a month or 3 mg IV once every 3 months. Women with fracture risk (femoral neck BMD T-score less than −3) had a significant reduction in nonvertebral fractures with ibandronate (9). However, A reduction in hip fracture risk has not been proven to be effective. It is advised to administer an IV infusion of zoledronate 5 mg once a year to treat postmenopausal women with PMO. In comparison to the placebo group, zoledronate significantly decreased the incidence of hip fractures (41%) and nonvertebral fractures (25%) in a major randomized experiment (5). In an additional trial, the frequency of recurrent clinical fractures (35% less) was significantly reduced in patients treated with zoledronic acid within 90 days following surgical repair of a hip fracture (10). About one-third of patients receiving their first intravenous injection experience an acute-phase reaction, or flu-like illness, which is the main side effect of this class of medications. Fever, soreness in the musculoskeletal system, and discomfort are typical symptoms of this reaction, which goes away in 2–3 days. Acetaminophen reduces the severity of symptoms and the incidence of this reaction (approximately 50%) (11). Another side effect is that potent antiresorptive medications can cause severe vitamin D deficiency (i.e., 25-hydroxy, vitamin D<25 nmol/L), and patients are predisposed to hypocalcemia (12). Therefore, before starting medication, patients must be assessed for sufficient calcium and vitamin D intake in addition to their serum 25-hydroxyvitamin D, calcium, phosphorus, and urine calcium excretion levels.

Because of their lengthy elimination half-life, bisphosphonates have the potential to build in the body. This accumulation may raise the risk of adverse responses because it excessively inhibits the conversion of bone. In theory, a “fallow period” could reduce the risk of bisphosphonate accumulation and maintain fracture prevention. Since risedronate has the lowest bone affinity, risk should be reviewed after a year by testing BMD and/or bone turnover markers; after 1–2 years for alendronate, 2–3 years for zoledronic acid, and after 1 year for risedronate. Typically, BMD is assessed every 2 years (4). After discontinuing medication, monitor bone mineral density. If bone density decreases by more than 6% over time, consider restarting bisphosphonate therapy or other medications.

### 2.2 Denosumab

### 2.3 DEXA hormone replacement therapy

The main strategies for reducing postmenopausal fractures in women 50 years of age and younger are estrogen therapy, estrogen + progestin (HT), or estrogen therapy (ET). Standard-dose MHT lowers fractures by 28% for hips, 35% for vertebral fractures, and 27% for other nonvertebral fractures, according to observational studies and randomized controlled trials (13). Women with prior hysterectomy should receive estrogen therapy alone (in the form of a transdermal patch or gel). Natural estrogens administered non-orally have the advantage of bypassing the first passage of hepatic effects and do not increase the risk of stroke, venous thromboembolism (VTE) or gallstones (14). Ethinyl estradiol, a synthetic estrogen, at an extremely low dose of 0.014 mg/day since it has been demonstrated to stop BMD from declining in postmenopausal women without inducing endometrial hyperplasia (15). Progestins are necessary for women who have an intact uterus since extended exposure to estrogen raises the risk of cancer and endometrial hyperplasia. Natural progesterone and medroxyprogesterone acetate (MPA) are progestins that are often utilized. Enough progestins taken in between 10 and 14 days of estrogen each month do not raise the risk of endometrial cancer (16). MHT also prevents genitourinary syndrome of menopause (GSM) and relieves symptoms such as hot flashes and night sweats caused by vasodilation (17). Hypotriglyceridemia, severe active liver illness, estrogen-sensitive breast or endometrial cancer in the past, inherited high-risk thromboembolism, and vaginal bleeding are possible complications of MHT. Along with mood changes, headaches, breast soreness, weight gain, bloating, nausea, and fluid retention, breakthrough bleeding (during the first 3–6 months) are common side effects. Thus, referrals for professional assessment are appropriate for women with complicated medical issues.

### 2.4 Raloxifene

Raloxifene is a selective estrogen receptor modulator (SERM) primarily indicated for women in the 50–59 age group. It binds to estrogen receptors and produces a mixed agonist–antagonist effect, with the effect of action varying from tissue to tissue, acting as an estrogen in some tissues but producing anti-estrogenic effects in others. Studies have demonstrated that raloxifene lowers the risk of vertebral fractures, but not the risk of hip fractures (18). According to a meta-analysis of the drug’s effects on BMD, after 2 years of raloxifene treatment, hip bone mineral density increased by 2.1% and lumbar spine bone mineral density increased by 1.8% (19). In an additional 3-year prophylactic raloxifene trial, 1,145 postmenopausal women 60 years of age or younger, with an average of 5 years of menopause and no osteoporosis, were randomized to the raloxifene group versus a placebo group. The results showed that raloxifene, at 30 mg/d to 150 mg/d, reduced bone turnover to normal premenopausal ranges and maintained spine, hip, and whole-body bone mineral density above baseline. Thus, raloxifene, at 60 mg/d, is recommended for the long-term prevention of postmenopausal bone loss (20).

### 2.5 Teriparatide

Teriparatide (TPTD), an agonist of the parathyroid hormone type 1 receptor, is made up of the first 34 amino acid fragments of the parathyroid hormone molecule. The degree of bone mass increases or decreases according to the manner of administration, as activation of parathyroid hormone receptors results in an increase in osteoclast and osteoblast activity. Teriparatide infusions administered continuously or once a day subcutaneously both promote bone growth, although they differ in their effects on bone mass and resorption. Continuous infusion resulted in a sustained increase in serum parathyroid hormone concentrations, which further led to increased bone resorption. In a study of teriparatide, a total of 1,637 postmenopausal women with previous vertebral fractures were treated with 20 or 40 μg of parathyroid hormone subcutaneously per day or placebo, and at the end of the study, daily injections of 20 and 40 μg of teriparatide per day resulted in an increase in spinal bone mineral density of 9 and 13%, respectively, and a reduction in the risk of new vertebral fracture of 65 and 69%, respectively, compared with placebo (21). In glucocorticoid-induced osteoporosis, the teriparatide group showed a more pronounced increase in BMD than alendronate and a significant reduction in vertebral (but not vertebral) fracture risk (22). Due to concerns about osteosarcoma, most countries limit the duration of teriparatide therapy to 24 months, and it is common practice to continue taking an antiresorptive drug (bisphosphonate or denosumab) (23). Nausea and headaches are frequently experienced with doses above 20 μg/day. Additionally, a small proportion of individuals may experience mild and temporary hypercalcemia after taking the medication. To address this side effect, adjusting the dosage may be necessary.

### 2.6 Romosozumab

Romosozumab is an anti-ossifying protein antibody that inhibits Wnt signaling in osteoblasts. Antibodies against sclerostin both promote the growth of new bone and prevent bone resorption. Romosozumab inhibits bone resorption via influencing the levels of osteoprotegerin. It is authorized for the management of severe osteoporosis in postmenopausal women who are extremely vulnerable to fractures. In a randomized controlled trial, in postmenopausal women with osteoporosis (excluding severe osteoporosis), the risk of new vertebral fracture and clinical fracture was significantly reduced by 1 year of romosozumab treatment compared with placebo (24). In an additional investigation involving postmenopausal women with severe osteoporosis, the experimental group received alendronate 70 mg orally once a week for 12 months after receiving romocizumab 210 mg subcutaneously once a month for 12 months, while the control group received alendronate 70 mg once a week for 24 months. Patients treated with romosozumab in addition to alendronate saw a considerably lower incidence of new vertebral, non-vertebral fractures than patients treated with alendronate alone (24). The rate of serious adverse cardiovascular events, including cardiac ischemia and cerebrovascular events, was higher in the romosozumab group than in the alendronate group in this trial (2.5% versus 1.9%) (0.8% versus 0.3%). The pathophysiology involves the expression of sclerostin in the smooth muscle of the aorta artery, which functions as a negative regulator of vascular calcification. Vascular calcification could result from sclerostin blockage. The investigation by Cosman et al. (24) did not get to this conclusion. In response to this contradictory result, Elena Tsourdi et al. conducted a further experimental analysis, the results of which showed that the incidence of serious cardiovascular adverse events due to romosozumab is controversial (25).

### 2.7 Isoflavone

Non-steroidal plant chemicals called phytoestrogens are found in nature and resemble estrogens. They may also have anti-estrogenic or estrogenic properties. When endogenous estrogens are sufficient in the body, phytoestrogens will act as “anti-estrogens.” It is only in postmenopausal “estrogen-free” conditions that the agonistic or “estrogen-like” effects of phytoestrogens predominate (26). Isoflavones are among the most estrogenically active plant derivatives, including genistein, soy flavonoids, glycoproteins, biotin A, and mancozebins, and their main sources are legumes. Isoflavones exert their effects via the traditional ER-mediated signaling route, while also stimulating intracellular pathways like MAPK, phospholipase C, and protein tyrosine kinases (27).

Accumulating evidence suggests that the anti-osteoporotic effects of flavonoids are closely related to their receptor selectivity. Most flavonoids, particularly isoflavones, demonstrate a preferential binding affinity for estrogen receptor *β* (ERβ). This is of particular significance because ERβ is abundantly expressed in bone tissue, whereas estrogen receptor *α* (ERα) predominates in estrogen-sensitive tissues such as the uterus and breast. Consequently, flavonoids can exert tissue-selective estrogenic actions, enhancing osteoblast differentiation and inhibiting osteoclast activity in bone, while exerting minimal stimulatory effects on reproductive tissues. Such receptor selectivity may reduce the risks typically associated with conventional hormone replacement therapy (HRT), including endometrial hyperplasia and an increased incidence of breast cancer (28). Both experimental and clinical studies have confirmed the differential distribution of ERα and ERβ across tissues, supporting the view that flavonoids may act as “SERM-like” agents with favorable bone-protective properties (29). Nevertheless, further research is warranted to clarify the extent to which ER subtype selectivity translates into clinically meaningful benefits for bone health.

Clinical trials have shown an increase in BMD and bone mineral content in women after 6 months of treatment with 90 mg/day of isoflavones (30). Both genistein (54 mg/day) and epoxyflavone (600 mg/day), according to a meta-analysis of randomized controlled trials, were safe for postmenopausal women and improved BMD outcomes. As a result, they may be used to prevent osteoporosis linked to menopause and as a supplemental or alternative therapy (31). Using data from 26 randomized controlled trials that included 2,652 women with low estrogen levels, the analysis found that isoflavones reduced moderate bone loss associated with the lumbar spine, femoral neck, and distal radius (32). The above research suggests that isoflavones may ameliorate menopause-associated imbalances in bone turnover, and protect bone density and bone strength. It is still debatable, though, whether soy isoflavone supplements are beneficial for treating and preventing osteoporosis in postmenopausal and perimenopausal women. According to a study by Levis et al. (29) menopausal women’s bone loss did not decrease after taking a 200 mg daily dosage of soy isoflavones for 2 years. According to a study by Kreijkamp-Kaspers et al. (33) After adding 25.6 g of soy protein for a year, postmenopausal women’s bone mineral density did not significantly change. Likewise, a different study found that giving postmenopausal women 110 mg of soy isoflavones daily for a year had no effect on bone turnover or prevention of postmenopausal bone loss (34). The anti-osteoporotic effects of flavonoids may be related to the balance between estrogen agonist and antagonist properties. In addition, it is possible that differences in study design may have led to differences in the reported study results.

### 2.8 Black Asclepias

Black Asclepias is a member of the Asclepias genus, also referred to as shengma and utilized as a traditional Chinese medicinal herb that is used worldwide. Triterpene glycosides, phenylacetones, nitrogenous chemicals, pigments, flavonoids, and 4-methyl steroids are the primary chemical elements of the plant. Among them, triterpene glycosides are its main bioactive components. Its mechanism of action in ameliorating osteoporosis is related to the exertion of antioxidant, anti-inflammatory, and estrogen-like effects (35). It preserves bone structure in the ovariectomized rat model by reducing bone resorption and inhibiting bone density loss (36). Furthermore, this study indicates that, in the ovariectomized rat model, estrogenic characteristics are exerted in adipose and bone tissue (mostly in osteoblasts), but not in the uterus, so demonstrating a selective estrogen receptor modulator (SERM) mechanism. In another animal study, black cohosh increased BMD and restored bone structure in de-ovulated animals (preventing decreases in BV/TV, Tb. Th. and Tb. N (37). García-Pérez et al. (38) showed that in menopausal women treated with black cohosh extract, urinary concentrations of N-terminal peptide (a marker of bone resorption) were decreased during the third month of treatment, whereas alkaline phosphatase (a marker of bone formation) increased. Nevertheless, a study by Bebenek et al. (39) findings demonstrated that there was no discernible impact of black cohosh extract on early postmenopausal women’s bone mineral density. The active ingredient in plant medicine is not a single chemical. Herbal extracts come in many forms, and their composition can vary depending on many parameters (such as standardization, culture, harvesting conditions, extraction methods, and extraction agents), which can lead to different efficacy and effects.

### 2.9 Co-administration

Co-administration refers to the administration of two types of medications sequentially or simultaneously. The romosozumab ARCH study assessed sequential therapy and found that, in comparison to alendronate monotherapy, romosozumab sequential alendronate therapy was more effective in avoiding fractures (40). A more frequent therapeutic situation involves treating a patient with an anti-resorptive drug, and when a fracture occurs, switching to a more potent intervention (such as anabolic medicine). Adding teriparatide to bisphosphonates or switching to teriparatide alone produced a greater increase in bone mineral density (BMD) than teriparatide alone did in an open-label trial involving women who had been on alendronate for more than 18 months (41). The transition from alendronate to romosozumab resulted in a greater increase in spinal BMD and prevented the notable loss of cortical bone in the hip that was seen with the changeover to teriparatide (40). While combined therapy led to a more significant increase in BMD, its more expensive treatment cost and associated drug side effects are something that needs to be evaluated in a comprehensive manner.

## 3 Conclusion

Osteoporosis needs lifetime care and is not a self-limiting condition; the goal of its treatment is to prevent fractures. Inadequate drug absorption and patient noncompliance following medication delivery are the most frequent causes of subpar BMD results. Which can be assessed by bone turnover markers after 2–3 months of treatment, and BMD needs to be reassessed after 2–6 years of treatment to evaluate the effectiveness of the medication. Over the past 30 years, efforts have been made to develop new treatments for osteoporosis. There are currently clinically accessible effective therapies to decrease bone resorption and boost bone growth. Future research needs to be more dedicated to the effective application of existing treatments. Large-scale, long-term observational studies are required to learn more about the safety and modes of action of currently available medications.
